# Supplementary material for: Modified Pearson correlation coefficient for two-color imaging in spherocylindrical cells
Source: BMC Bioinformatics. 2018 Nov 16;19:428. doi: 10.1186/s12859-018-2444-3 (PMC6240329; doi:10.1186/s12859-018-2444-3)
Supplement: Supplementary file 1 — SI Text S1. Two additional cases of anti-correlation in 3D and in 2D. SI Text S2. Example of positive correlation. SI Text S3. Materials and Methods. SI Text S4. Determining cell width and length. SI Figures (S1-S8) and Captions. Table S1. Variation of MPCC with number of pixels Np and with mean occupancy per pixel for the experimental RNAP (green) and HU (red) image matrices. (DOCX 2165 kb) [file 12859_2018_2444_MOESM1_ESM.docx]

**Additional file 1**

**SI Text**

**SI Text S1. Two additional cases of anti-correlation in 3D and in 2D**

We tested two additional cases (Case II and III) of images sampled from perfectly anti-correlated distributions in both 3D and 2D, shown in Fig. S1. In these two cases, both the red and green channels contain 10,000 molecules. The molecules constituting **R** and **G** are a subset of a larger number of molecules distributed randomly in a spherocylinder. In Case II, the two end cap regions are occupied by red molecules and the central region of the spherocylinder is occupied by green molecules. The volume occupied by red molecules is greater than that occupied by green molecules. In Case III, the red molecules are confined to the leftmost 2/3 of the spherocylinder and the rest of the volume is occupied by green molecules. The volume occupied by red molecules is greater than that of the green molecules. In both Cases II and III, the red and green molecules never occupy the same region of space in 3D or 2D, the hallmark of perfect anti-correlation. For all the cases including case I described in the main text, the linear anti-correlation relationship is demonstrated by the scatter plot of $\hat{\Delta}_{ij}^{R}$ vs $\hat{\Delta}_{ij}^{G}$ (Fig. S1*B*). The calculated MPCC in these cases is –0.99, approaching the expected value of –1 within the limits of noise. However, the calculated PCC is –0.49, significantly underestimating the degree of anti-correlation.

**SI Text S2. Example of positive correlation**

An example of perfect positive correlation is presented in the main text. A detailed comparison of MPCC and PCC applied to this case is provided in Fig. S2.

**SI Text S3. Materials and Methods**

**Strain construction**

For determining cell radius and length, we imaged the photoactivatable fluorescent protein Kaede (1, 2), which was expressed from a tetracycline inducible plasmid pASK IBA3 plus (IBA). *E. coli* DH5α strain expressing Kaede was constructed as described by Bakshi *et al* (3). This vector was then transformed into VH1000 using the TSS protocol and selected against ampicillin resistance. For imaging RNAP, we used an *E. coli* VH1000 strain that endogenously expresses the RNAP β’ subunit translationally fused with yGFP. Construction of the *rpoc*::*ygfp* strain was described before by Bakshi *et al*. The fluorophore yGFP is a variant of GFP (4). It absorbs green light (λ_max_ = 514 nm) and fluoresces green-yellow (λ_max_ = 533 nm) (5).

The DNA binding protein HU – PAmcherry used for imaging the nucleoid was expressed from a plasmid. The gene *hupA* (HU) was amplified from VH1000 using the primers (primers, 5’-ATCGGATCCCGCCGCCGCCTTAACTGCGTC-3’and 5’-GATCGAATTCTA GGAGGTATTCACATGAACAAGACTC-3’). The amplified *hupA* gene with restriction sites for EcoRI and BamHI was inserted into pASK IBA3 plus (IBA). Then *PAmcherry* was amplified using primers (primers, 5’ -GATCAAGCTTTTACTTGTACAGCTCGTCCATGCC-3’ and
5’-GATCGGATCCGTGAGCAAGGGCGAGG-3’). The amplified *PAmcherry* with restriction sites for BamHI and HindIII was inserted into the *hupA* vector. This vector was transformed into VH1000 using the TSS protocol and selected against ampicillin resistance.

**Cell growth and preparation for imaging**

Bulk cultures from frozen glycerol stock solution and subcultures for imaging were grown at 30°C with continuous shaking in 100 µg/mL ampicillin containing EZ rich, defined medium (“EZRDM”), which is a MOPS-buffered solution with supplemental metal ions (M2137; Teknova), glucose (2 mg/mL), supplemental amino acids and vitamins (M2104; Teknova), nitrogenous bases (M2103; Teknova), 1.32 mM K_2_HPO4, and 76 mM NaCl.

When cells had grown to midlog phase, anhydrotetracycline was added to a final concentration of 25 nM to induce the expression of the desired protein. After 5 min of induction, the cells were centrifuged and resuspended in fresh EZRDM with 100 µg/mL ampicillin to remove the inducer. The cells were then incubated again in growth medium for 15 min at 30°C to enable maturation of the respective fluorescent protein prior to imaging.

**Superresolution imaging of live *E. coli* cells**

Fluorophore-labeled cells were grown overnight with shaking at 30°C in EZRDM. Subcultures were made by diluting the stationary phase culture at least 1:100 into 2 mL of fresh EZRDM. Subcultures were grown to exponential phase (OD = 0.2–0.6 at 600 nm), upon which the culture was placed in CoverWell Perfusion Chamber Gaskets (Invitrogen, Carlsbad, CA) on a polylysine-coated coverslip. The volume of the closed chamber is ~ 150 µL. We allowed ~2 min for the cells to adhere to the coverslip, then replaced the liquid in the chamber with fresh, aerated medium to rinse away the non-adhered cells. Live cell imaging was carried out at 30°C for no longer than 30 min after plating. During that time, cells continue to grow.

Cells were imaged using an Eclipse Ti inverted microscope (Nikon Instruments, Melville, NY) equipped with an oil immersion objective (CFI Plan Apo Lambda DM 100x Oil, 1.45 NA; Nikon Instruments), a 1.5x tube lens, and the Perfect Focus System (Nikon Instruments, Melville, NY).

For imaging Kaede, we used a fast back-illuminated electron-multiplying charge-coupled device (EMCCD) camera with 128 x 128 pixels of 24 x 24 μm each (iXon DV-860; Andor Technology, South Windsor, CT). Each pixel corresponds to 160 x 160 nm^2^ at the sample (150x overall magnification). For acquisition of fast diffusing Kaede, all data were collected at a frame rate of 485 Hz, with exposure time within each frame of 2 ms. The Kaede was photoactivated with a 405-nm diode laser (CrystaLaser, Reno, Nevada) and subsequently imaged with a 561-nm laser (Sapphire 561 CW lasers; Coherent, Bloomfield, CT). The 405-nm power density at the sample was 4-15 W/cm^2^. The photoactivation laser remained on throughout imaging. Power density of the 561 nm laser was kept at ~2 kW/cm^2^. The probe laser was on continuously in the
2 ms/frame tracking experiments. To minimize the phototoxic effect of the laser, we collected data for <5 s per cell. The Kaede emission was collected through a 617/73 bandpass filter (bright line 617/73-25; Semrock, Rochester, NY).

Images of cells that express RNAP β’ – yGFP along with HU – PAmcherry were recorded by a back – illuminated EMCCD camera with 512 x 512 pixels of 16 μm x 16 μm pixels (iXon DV – 887; Andor Technology, CT, USA). Each pixel corresponds to 105 x 105 nm^2^ at the sample with an overall magnification of 150x. All images were collected at a frame rate of 31.2 Hz, with an exposure time within each frame of 30 ms. The RNAP β’–yGFP molecules were illuminated with the 514 nm laser with a power density of ~2 kW/cm^2^. Images were acquired only when the rate of return of molecules to the fluorescent state becomes small enough that at most 3-4 copies per camera frame are fluorescent in each cell, enabling single molecules to be distinguished from each other. To minimize the phototoxic effect of the laser, we collected data for <35 s per cell. The HU–PAmcherry molecules were photoactivated with 405 nm diode laser and subsequently imaged with the 561 nm laser. The 405-nm power density at the sample was 6 - 10 W/cm^2^ to ensure only 3 - 4 molecules of HU–PAmcherry are fluorescent per camera frame per cell. The power density of the 561 nm laser was kept at ~ 2 kW/ cm^2^ and the laser was continuously on during the 30 ms/frame imaging experiments. Similarly, the cells were imaged for HU–PAmcherry for <35 s per cell to ensure minimum cell damage and normal cell growth during the imaging period. We used polychroic ZT405-514-561rpc (Chroma, VT, USA). The fluorescence of RNAP β’–yGFP and HU–PAmcherry were isolated using triple band pass filter ZET442/514/561m (Chroma, VT, USA). We also added a 525 nm long pass filter (ET525lp, Chroma, VT, USA) to block the shorter wavelength band allowed by the used polychroic. To minimize leakage of emission of yGFP into the red channel, we first imaged S2–yGFP which results in photobleaching of majority of yGFP molecules.

**Single molecule image analysis**

Images were analyzed using a MATLAB GUI developed in our lab (3). Images were smoothed and filtered to obtain a zero-based image. Bright spots were located with pixel level accuracy by a peak-ﬁnding algorithm that ﬁnds local intensity maxima within an image. A user-defined intensity threshold was used as the minimum brightness of a pixel from single molecule trajectories. It is carefully set by the user so that it will not be so high as to cut a long trajectory short or so low as to include background noise.

Centroids of the bright spots of RNAP β’-yGFP and HU-PAmcherry were calculated from a 7 x 7 pixel square centered on the local maxima determined by the peak-finding algorithm. Since fast diffusing Kaede are blurred extensively, we use an 11 x 11 pixel square centered on the local maxima to calculate the centroid. As the images are asymmetrically blurred due to diffusion during the frame time, we calculate the centroid of the bright spots instead of using Gaussian ﬁtting. This centroid analysis is fast and easily implemented for analysis of Monte Carlo modeling results as well. The (*x*, *y*) positions of the centroid are utilized to generate 2D localization probability density maps. For single-molecule tracking analysis, the (*x*, *y*) positions of the centroid are stored and connected into trajectories using a modiﬁed MATLAB version of the tracking program written by Crocker and Grier (6). These trajectories will be used in determination of mean diffusion coefficient.

**Determination of localization error of imaged molecules**

The MSD as a function of lag time * *is given by $\mathrm{MSD}\left( \tau\right)= <{(\boldsymbol{r}\left( t+\tau\right)-\boldsymbol{r}\left( t \right))}^{2}>$, where ***r***(*t*) is the two-dimensional location of the particle at time *t*,  is the lag time, and the average is taken over all times *t* and over many trajectories. We chose trajectories that lasted for at least seven frames in order to minimize studying of spurious molecules. Tracks longer than seven frames were truncated to seven frames. Suppose the best fit to the first two experimental points of a two dimensional mean-square displacement plot is given by the equation MSD () = *a* + *b*τ, with *b* the slope and *a* the extrapolated intercept at lag time  = 0. Then the best estimate of the diffusion coefficient is *D* = *b*/4 and the best estimate of the dynamic localization error is
 = ½ (*a* + 4*Dt*_E_/3)^1/2^, where *t*_E_ is the exposure time per camera frame (7).

For RNAP β’-yGFP, using 685 trajectories we determined the diffusion coefficient to be 0.064 ± 0.004 μm^2^-s^-1^ and σ_RNAP_ = 38 nm (Fig. S7). For HU-PAmcherry, using 1180 trajectories we determined the diffusion coefficient to be 0.26 ± 0.04 μm^2^-s^-1^ and σ_HU_ = 60 nm in (Fig. S7).

**SI Text S4. Determining cell width and length**

The cytoplasmic radius varies little from cell to cell To determine the cell radius *r*, we imaged photoactivable Kaede molecules, which are believed to spatially distribute homogenously in cytoplasmic volume (3). We used data from 15 cells under same growth conditions used for imaging cells expressing RNAP β’-yGFP and HU-PAmcherry. MSD(τ) plot for Kaede imaged with an exposure time of 2 ms is shown in Fig. S6*A*. We chose 709 trajectories that lasted for at least seven frames. Tracks longer than seven frames were truncated to seven frames to avoid overcounting. The mean diffusion coefficient for Kaede was measured to be *D*_Kaede_ = 4.77 ± 0.21 μm^2^-s^-1^. For *a* = 0.025 μm^2^, *D* = 4.77 μm^2^-s^-1^, and *t*_E_ = 2 ms,the dynamic localization error is estimated to be σ_Kaede_ = 97 nm.

To estimate the cytoplasmic radius *r*, we then simulated Kaede diffusion using
*D*_Kaede_ = 5.0 μm^2^-s^-1^ and σ_Kaede_ = 97 nm in uniformly filled spherocylinders of varying radii, seeking a match to the experimental transverse spatial distribution. The model spherocylinders had cylindrical length of 3.5 μm. At t = 0, 15,000 particles were randomly distributed within the cell volume. Each particle undergoes a random walk independent of other particle positions. To model each 2-ms camera image, three-dimensional microtrajectories (1000 steps of 2 μs each) were generated. At each time step, each particlechooses a displacement in each of three Cartesian directions. These displacements are chosen from a Gaussian distribution whose standard deviation corresponds to the three-dimensional diffusion coefficient D_Kaede_ = 5 μm^2^-s^-1^. In the rare event that a particle attempts to step outside of the cell boundaries, the displacement for that microstep is taken to be zero. The location of each particle during each camera frame is obtained as the centroid of the model microtrajectories in order to mimic the analysis procedure used for the experimental images. The appropriate dynamic localization error σ_Kaede_ was then applied to each centroid location in both *x* and *y* coordinates by sampling a Gaussian distribution with standard deviation  =  nm By adding the error vector to the centroid position we obtain the model “measured” location for each 2 ms camera frame. The *x* and *y* coordinates of each measured location are stored for further analysis. For the next model camera frame, each particle continues to make microsteps in 3D starting from the endpoint of the previous camera frame. Only the particles in the central cylindrical region are used to generate the simulated radial distribution; the endcaps are deleted.

For each of 15 cells, we found the best fit of the experimental distribution to the spherocylindrical model in a least-squares sense by varying the model radius in increments of
10 nm. A single-cell experimental radial distribution of 1725 Kaede locations, excluding the endcap regions of the cell, is shown in Fig. S6*B*. The best-fit simulated radial distribution had *r* = 390 nm (Fig. S6*B*). Averaged over 15 cells, the mean cytoplasmic radius is
*r* = 412 ± 22 nm (± 1.

For measuring each cell length *L_cell_*, we scattered the localizations of the molecules imaged in the cells in the chosen length bin as shown in Fig. S6*C*. The points at which the scattered distributions begin to curve mark the beginnings of the hemispherical endcap regions and the ends of the cylindrical region. The error in determination of the inflection point is in the orders of ~50 nm which is significantly smaller than the measured cell length. The distance between these two inflection points is the length of the cylindrical region of the cell (*L_cyl_*). The measured tip-to-tip cell length is then *L_cell_* = *L_cyl_* + 2*r*.

**SI Figures and Captions**


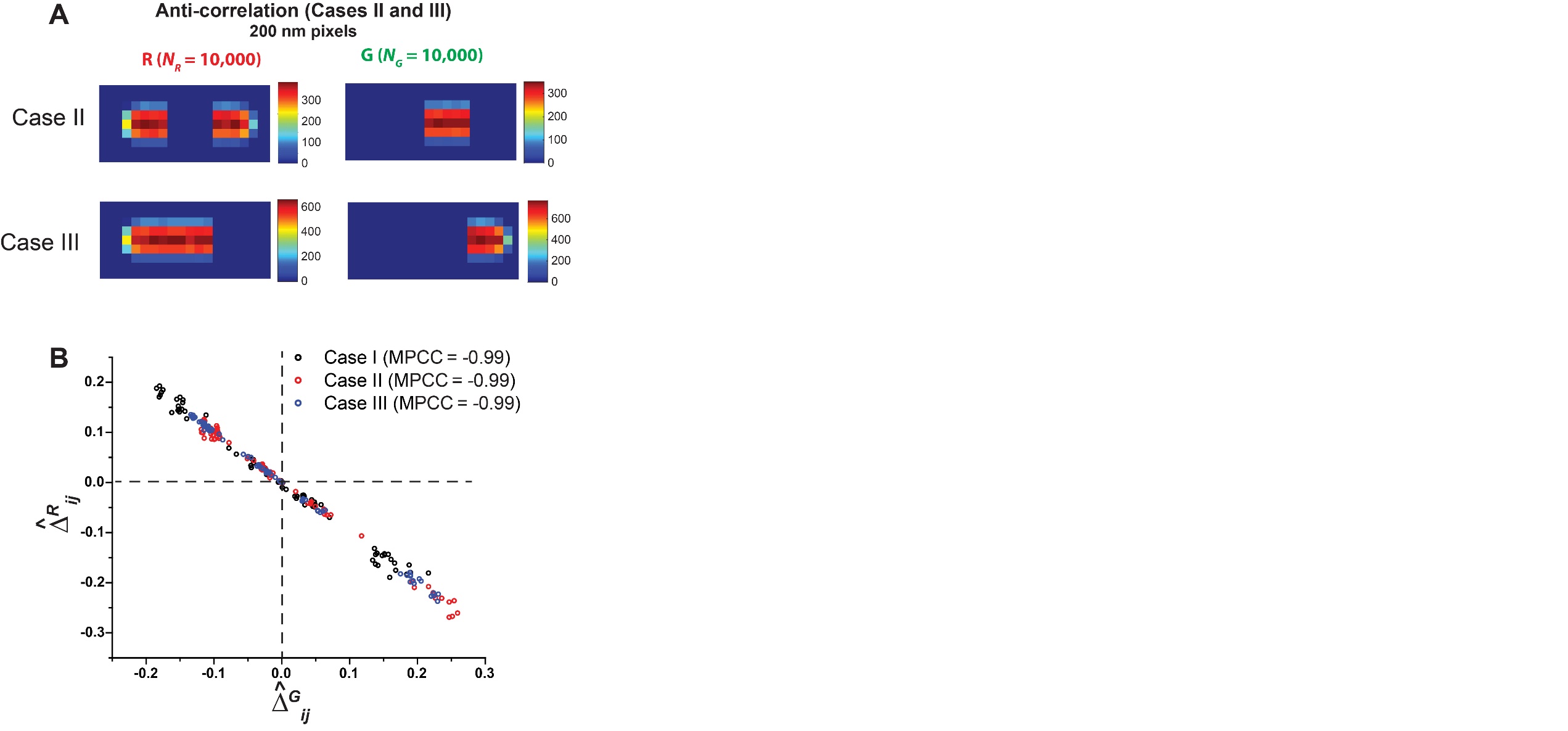


**Figure S1.** Additional cases of images sampled from perfectly anti-correlated distributions in 3D and 2D. In each case, there are ~10,000 red and ~10,000 green molecules, with 200 nm pixels. **(A)** *Top*: Case II. *Bottom*: Case III. The color of each pixel indicates the number of molecules as shown in the color bar. **(B)** Scatter plot of individual difference elements, $\hat{\Delta}_{ij}^{R}$ vs. $\hat{\Delta}_{ij}^{G}$ for Case I described in main text (*Black*), Case II (*Red*), and Case III (*Blue*). The calculated MPCC is –0.99 in all three cases.

**
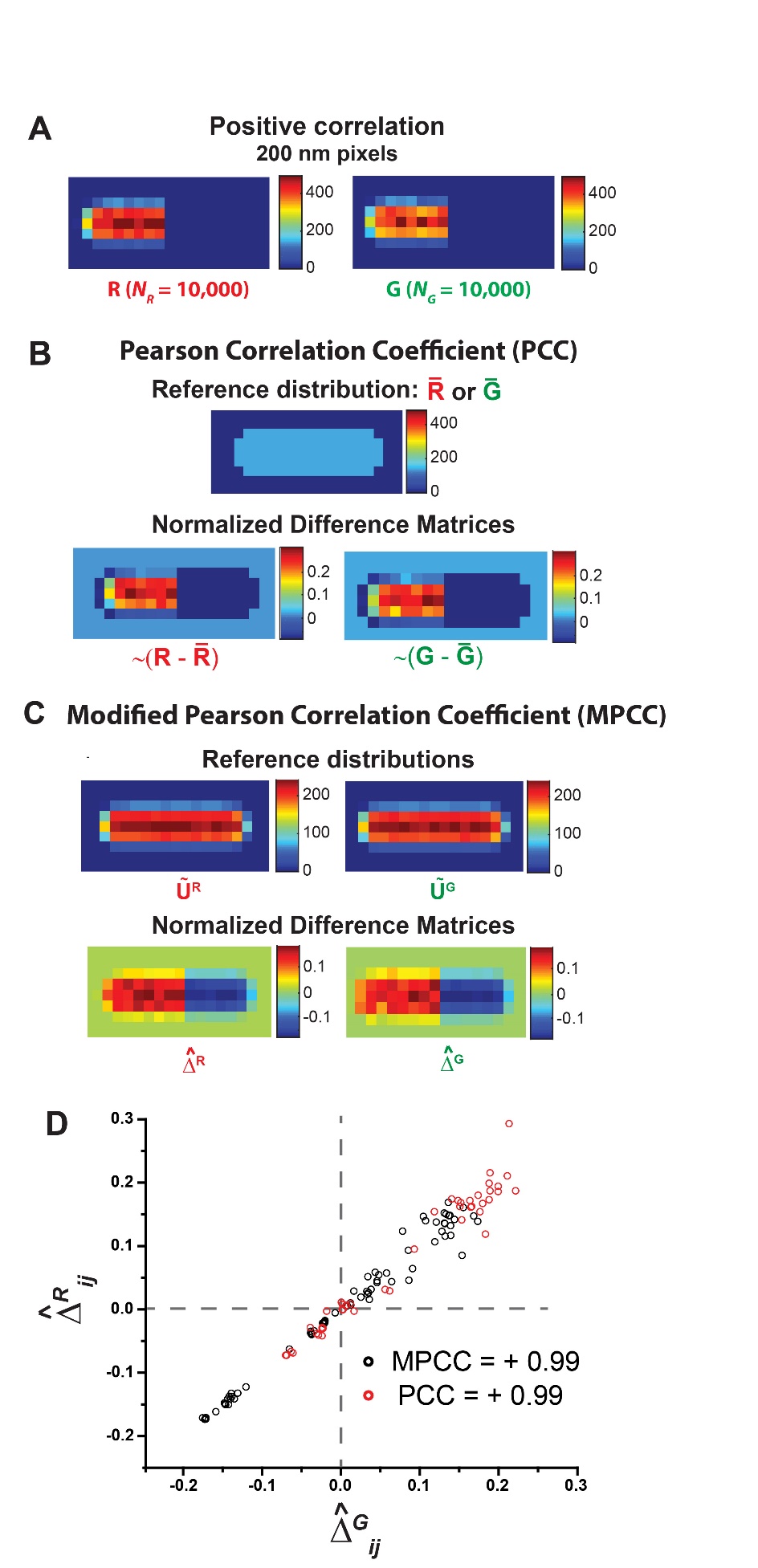
**

**Figure S2.** Scheme for calculating PCC and MPCC for two representative images **R** and **G** that are positively correlated in both 3D and 2D. **(A)** Images **R** and **G** with pixels of size 200 nm, each containing ~10,000 molecules. The color of each pixel is indicative of the number of molecules as shown in the adjacent color bar. **(B)** Standard PCC calculation. *Top*: Reference distribution $\bar{\mathbf{R}}$ or $\bar{\mathbf{G}}$, with same individual pixel counts, that is subtracted from images **R** or **G**. *Bottom*: Normalized difference matrices $\boldsymbol{\sim(R-}\bar{\mathbf{R}}\mathbf{)}$ and $\boldsymbol{\sim(G-}\bar{\mathbf{G}}\mathbf{)}$ obtained after subtraction. The Frobenius inner product of these two difference matrices gives the PCC. **(C)** Modified PCC calculation. *Top*: Reference distribution ${\tilde{\mathbf{U}}}^{\mathbf{R}}$or ${\tilde{\mathbf{U}}}^{\mathbf{G}}$, which are 2D projections of 3D random distribution in a spherocylinder, that is subtracted from images **R** or **G**. *Bottom*: Normalized difference matrices ${\hat{\boldsymbol{\Delta}}}^{\mathbf{R}}$and ${\hat{\boldsymbol{\Delta}}}^{\mathbf{G}}$ obtained after subtraction. The Frobenius inner product of these two difference matrices gives the MPCC. **(D)** Scatter plot of individual difference elements, $\hat{\Delta}_{ij}^{R}$ vs. $\hat{\Delta}_{ij}^{G}$, for PCC (*Red*) and MPCC (*Black*). The resulting MPCC and PCC values are both +0.99.

**
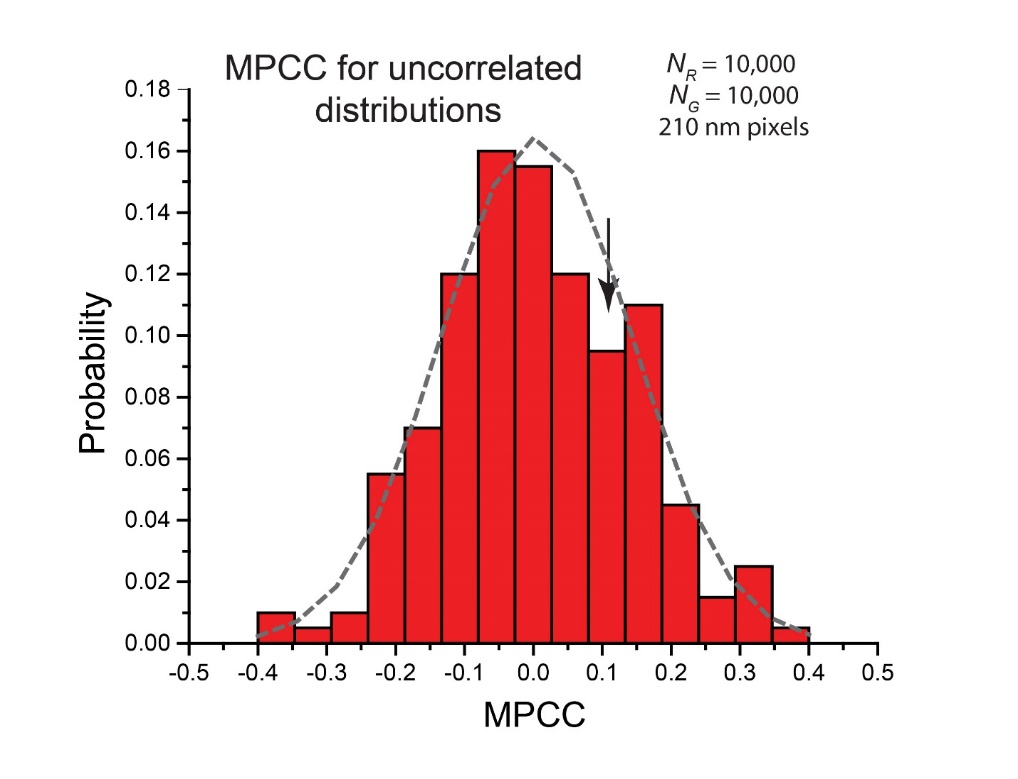
**

**Figure S3.** Probability distribution of 200 MPCC calculated for sets of **R** and **G** matrices formed from 2D projections of uncorrelated, random 3D distributions. Each **R** and **G** matrix contains 10,000 molecules and uses 210 nm pixels. The best-fit Gaussian curve (*dashed grey line*) has mean <MPCC> = 0.0041 and standard deviation σ_MPCC_ = 0.13. The arrow shows the bin for the value MPCC = +0.14, the result for the sample calculation in the main text (Fig. 3).

**
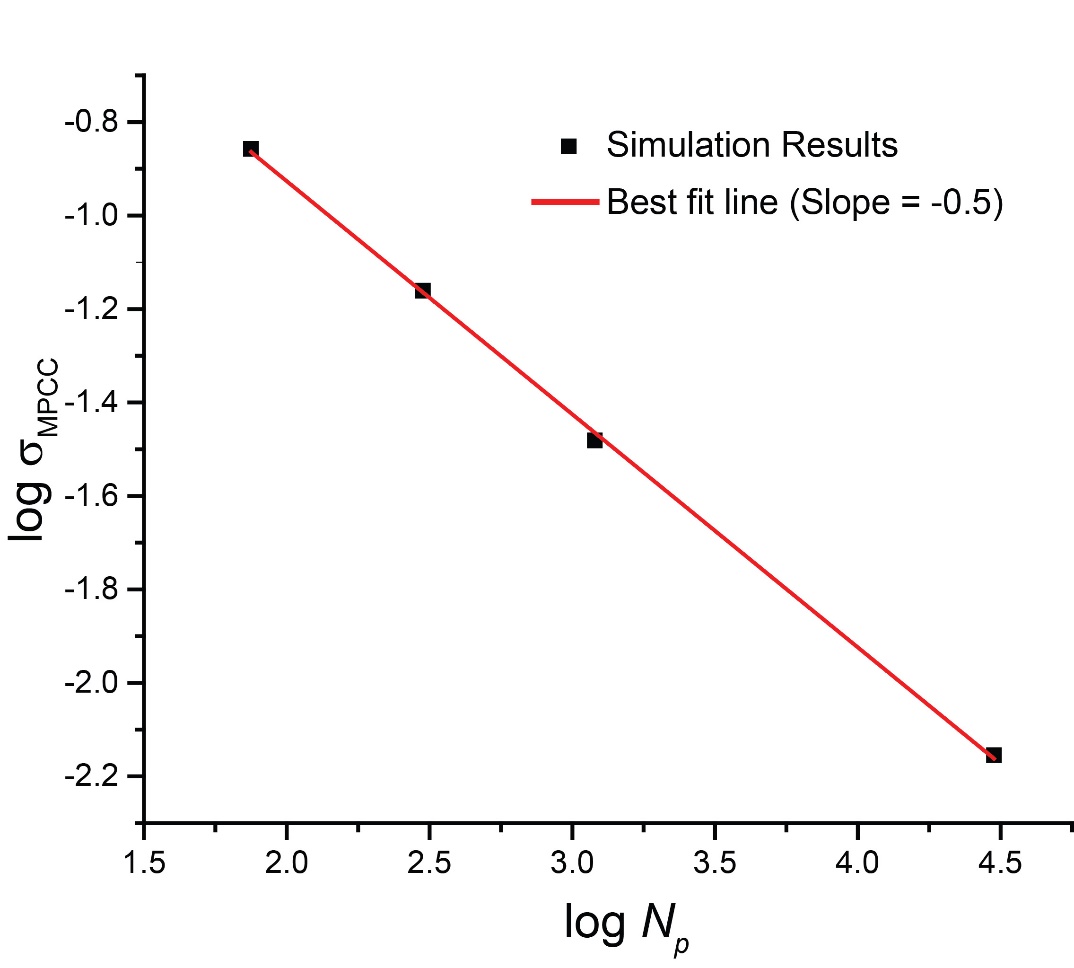
**

**Figure S4.** Dependence of σ_MPCC_ (Fig. S3) on the total number of pixels (*N_p_*) for uncorrelated **R** and **G** matrices. The number of red and green molecules is held constant at ~10,000 each. Best-fit line to log σ_MPCC_ vs. log *N_p_* has slope –0.49.


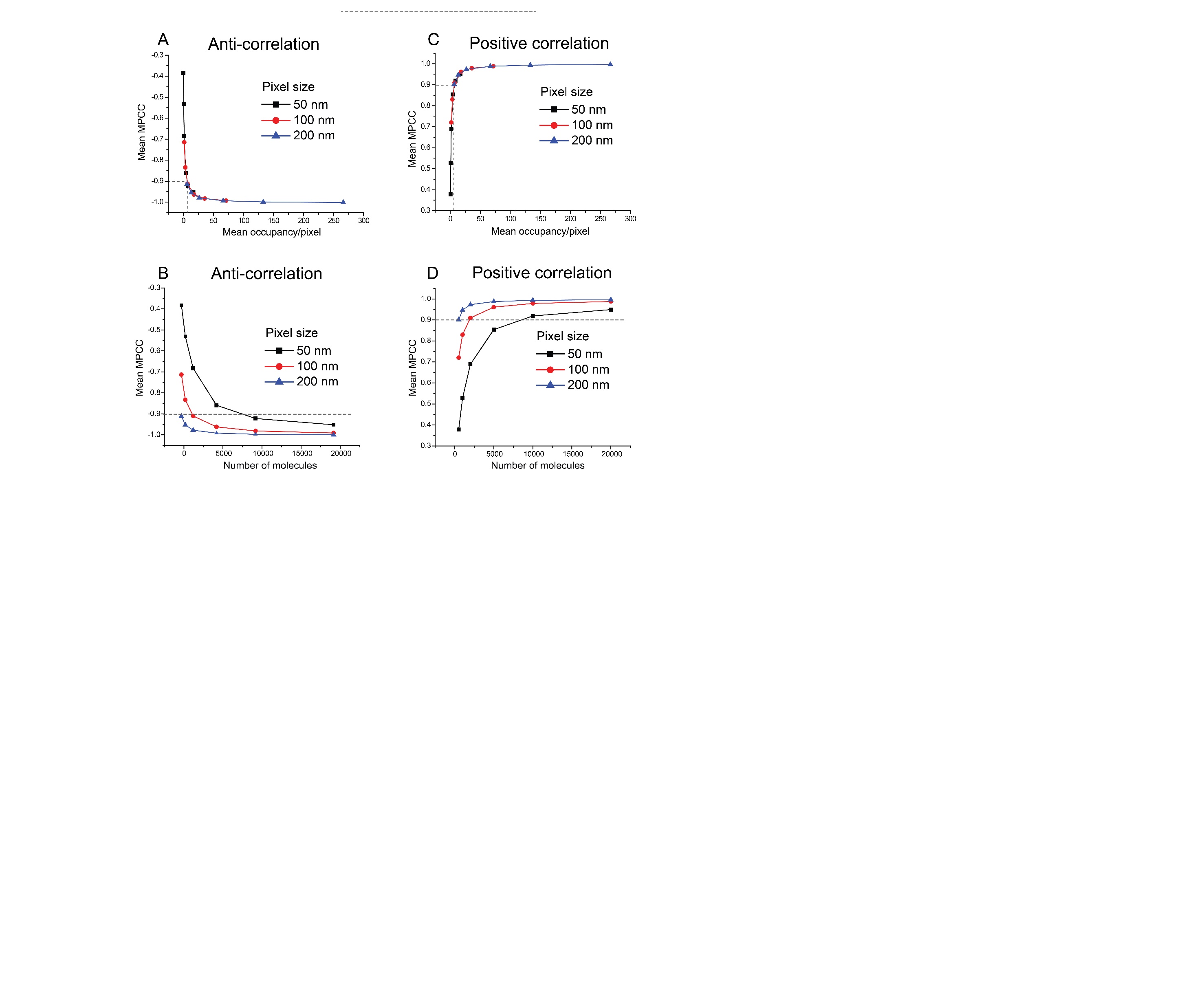


**Figure S5.** Variation of mean MPCC (calculated from 200 trials as in Fig. S3) with number of pixels *N_p_* and with total number of red and green molecules in the image matrices **R** and **G**. Models of perfect anti-correlation (as in Fig. 2) and perfect positive correlation (as in Fig. S2) were tested. **(A)** Anti-correlated images **R** and **G**. <MPCC> vs mean occupancy/pixel for three different pixel sizes as shown: 50 nm (*N_p_* = 1178), 100 nm (*N_p_* = 279), and 200 nm (*N_p_* = 77).
**(B)** Same data as in **(A)**, now plotted vs the number of red and green molecules. **(C)** Positively correlated images **R** and **G**. <MPCC> vs mean occupancy/pixel for three different pixel sizes as shown: 50 nm (*N_p_* = 1178), 100 nm (*N_p_* = 279), and 200 nm (*N_p_* = 77). **(D)** Same data as in **(C)**, now plotted vs the number of red and green molecules.

**
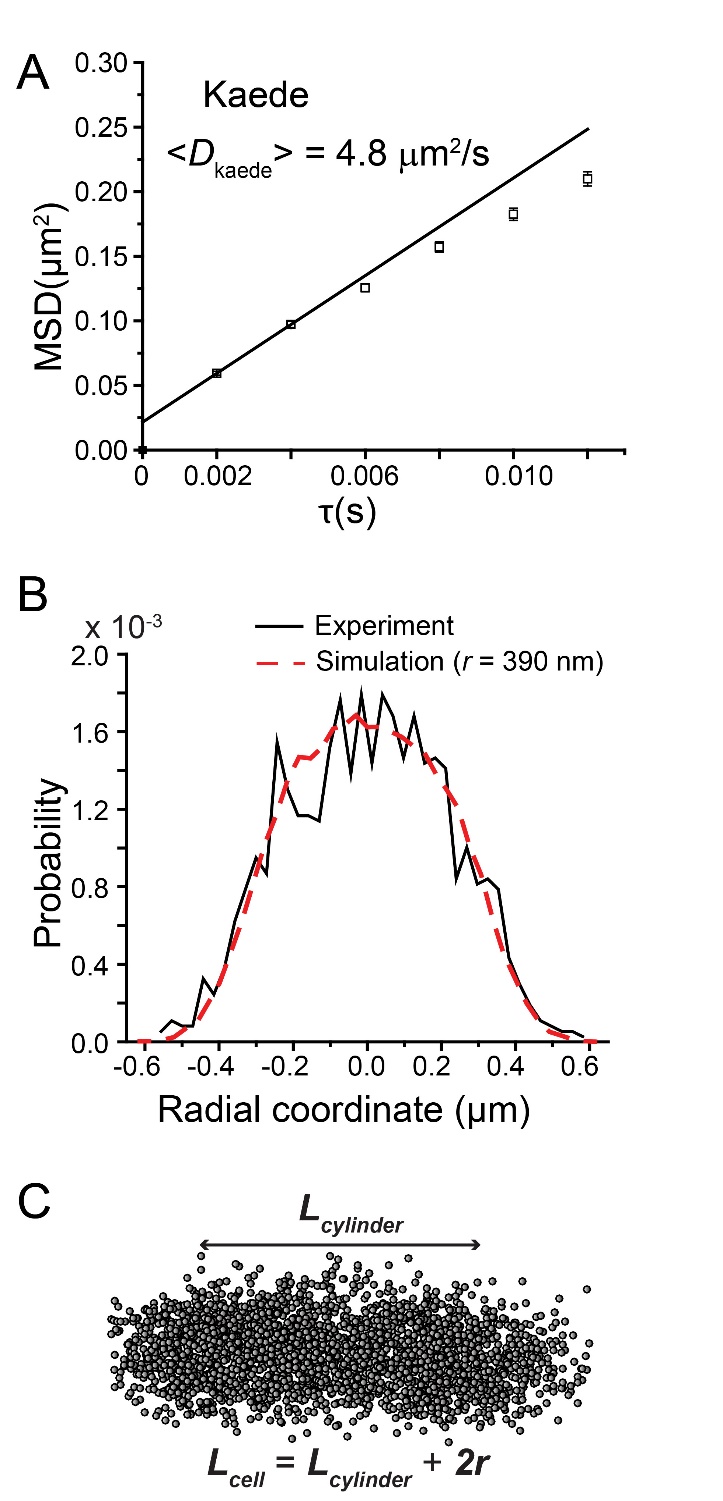
**

**Figure S6. (A)** Mean-square displacement plot, MSD(τ), for Kaede under imaging conditions described in **SI Text S3**. Trajectories are truncated to six steps; error bars are ± one standard error of the mean. The curvature is likely due to confinement. Line drawn through first two data points yields the estimated mean diffusion coefficient <*D_Kaede_*> = 4.77 µm^2^/s. **(B)** (*Black*) An example experimental single-cell radial distribution of Kaede molecules within the straight cylindrical region of the cell (endcaps excluded). (*Dashed red*) Simulated radial distribution of homogenously distributed molecules in a 3D spherocylinder. Best-fit radius is *r* = 390 nm.
**(C)** Scatter plot of imaged Kaede molecules in a single cell. *L_cylinder_* is taken as the length of the cylindrical region, as judged by eye. Tip-to-tip length *L­_cell_* = *L_cylinder_* + 2*r.*

**
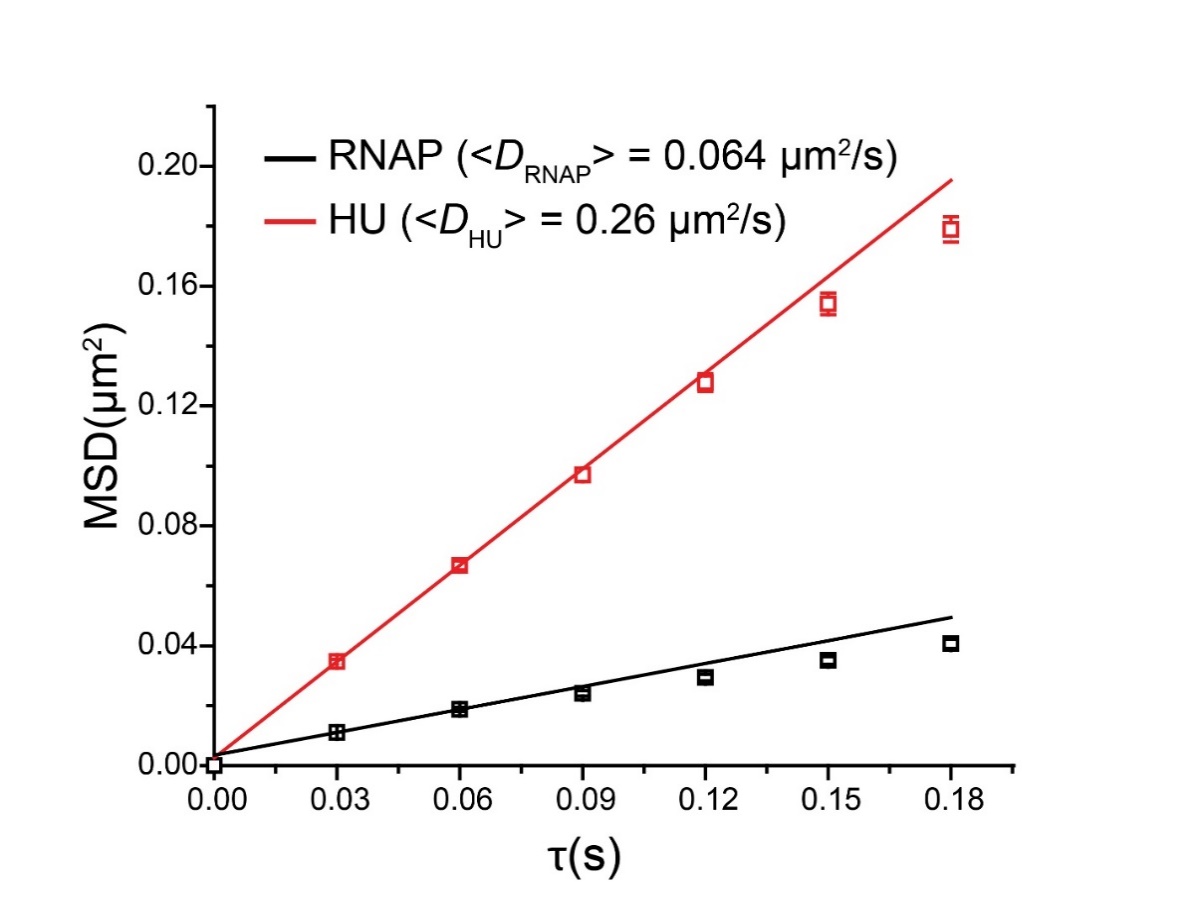
**

**Figure S7.** Mean-square displacement plots, MSD(τ), for HU–PAmcherry (*red squares*) and for RNAP–YFP (*black squares*). Imaging conditions described in **SI Text S3**. Trajectories are truncated to six steps; error estimates are ± one standard error of the mean. Lines drawn through first two data points yield estimated mean diffusion coefficients <*D_HU_*> = 0.26 µm^2^/s and <*D*_RNAP_> = 0.064 µm^2^/s.

**Table S1.** Variation of MPCC with number of pixels *N_p_* and with mean occupancy per pixel for the experimental RNAP (green) and HU (red) image matrices. Total number of imaged RNAP and HU molecules are 6570 and 8436 respectively. N_RNAP_ and N_HU_ were varied by randomly deleting red and green molecules. Pixel sizes are 200 nm, 100 nm and 50 nm in A), B) and C) respectively.

**A) Pixel size = 200 nm, *N_p_* = 77**

| N_RNAP_ | N_HU_ | Mean occupancy/pixel | MPCC |
| --- | --- | --- | --- |
| 6570 | 6570 | 85.32 | 0.51 |
| 5000 | 5000 | 64.93 | 0.50 |
| 4000 | 4000 | 51.95 | 0.50 |
| 2000 | 2000 | 25.97 | 0.45 |
| 1000 | 1000 | 12.99 | 0.38 |
| 500 | 500 | 6.49 | 0.30 |

**B) Pixel size = 100 nm, *N_p_* = 279**

| N_RNAP_ | N_HU_ | Mean occupancy/pixel | MPCC |
| --- | --- | --- | --- |
| 6570 | 6570 | 23.55 | 0.41 |
| 5000 | 5000 | 17.92 | 0.39 |
| 4000 | 4000 | 14.34 | 0.37 |
| 2000 | 2000 | 7.17 | 0.29 |
| 1000 | 1000 | 3.58 | 0.21 |
| 500 | 500 | 1.79 | 0.14 |

**C) Pixel size = 50 nm, *N_p_* = 1178**

| N_RNAP_ | N_HU_ | Mean occupancy/pixel | MPCC |
| --- | --- | --- | --- |
| 6570 | 6570 | 5.58 | 0.28 |
| 5000 | 5000 | 4.24 | 0.24 |
| 4000 | 4000 | 3.39 | 0.21 |
| 2000 | 2000 | 1.70 | 0.14 |
| 1000 | 1000 | 0.85 | 0.08 |
| 500 | 500 | 0.42 | 0.04 |


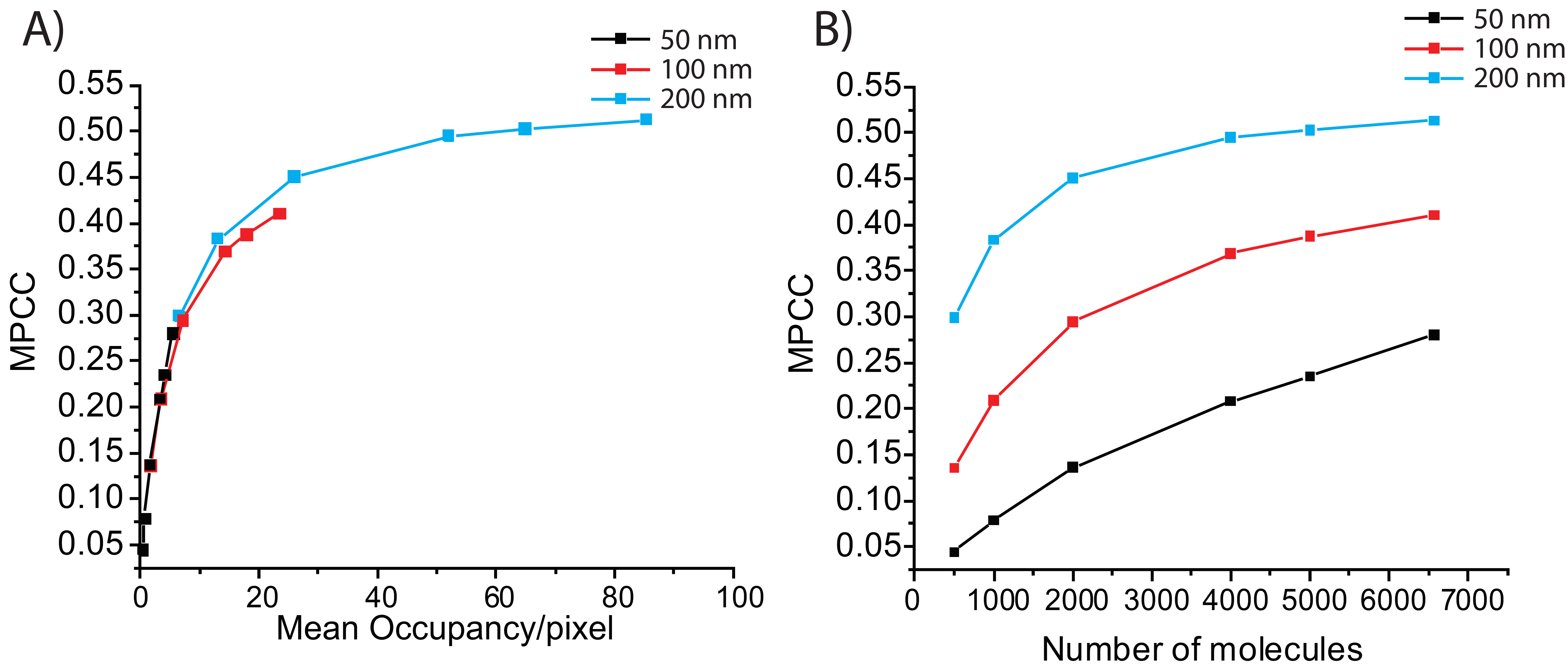


**Figure S8.** Plots of the data in Table S1. Variation of MPCC with number of pixels *N_p_* and with number of molecules for the experimental RNAP (green) and HU (red) image matrices. Total number of imaged RNAP and HU molecules are 6570 and 8436 respectively. N_RNAP_ and N_HU_ were varied by randomly deleting red and green molecules. **(A)** MPCC vs mean occupancy/pixel for three different pixel sizes as shown: 50 nm (*N_p_* = 1178), 100 nm (*N_p_* = 279), and 200 nm (*N_p_* = 77). **(B)** Same data as in **(A)**, now plotted vs the total number of red and green molecules. Mean occupancy per pixel is evidently the controlling parameter in the approach of MPCC to its asymptotic limit.

**Supporting References**

1. Ando R, Hama H, Yamamoto-Hino M, Mizuno H, Miyawaki A. An optical marker based on the UV-induced green-to-red photoconversion of a fluorescent protein. Proc Natl Acad Sci U S A. 2002;99(20):12651-6.

2. Hayashi I, Mizuno H, Tong KI, Furuta T, Tanaka F, Yoshimura M, et al. Crystallographic Evidence for Water-assisted Photo-induced Peptide Cleavage in the Stony Coral Fluorescent Protein Kaede. J Mol Biol. 2007;372(4):918-26.

3. Bakshi S, Bratton Benjamin P, Weisshaar James C. Subdiffraction-Limit Study of Kaede Diffusion and Spatial Distribution in Live *Escherichia coli*. Biophys. J. 2011;101(10):2535-44.

4. Bakshi S, Siryaporn A, Goulian M, Weisshaar JC. Superresolution Imaging of Ribosomes and RNA Polymerase in Live *Escherichia coli* Cells. Mol Microbiol. 2012;85(1):21-38.

5. Nielsen HJ, Ottesen JR, Youngren B, Austin SJ, Hansen FG. The *Escherichia coli* chromosome is organized with the left and right chromosome arms in separate cell halves. Mol Microbiol. 2006;62(2):331-8.

6. Crocker JC, Grier DG. Methods of Digital Video Microscopy for Colloidal Studies. J Colloid Interface Sci. 1996;179(1):298-310.

7. Michalet X. Mean square displacement analysis of single-particle trajectories with localization error: Brownian motion in an isotropic medium. Phy Rev E. 2010;82(4):041914.
